# Are Kazakhstani Women Satisfied with Antenatal Care? Implementing the WHO Tool to Assess the Quality of Antenatal Services

**DOI:** 10.3390/ijerph15020325

**Published:** 2018-02-13

**Authors:** Marzhan A. Dauletyarova, Yuliya M. Semenova, Galiya Kaylubaeva, Gulshat K. Manabaeva, Bakytkul Toktabayeva, Maryash S. Zhelpakova, Oxana A. Yurkovskaya, Aidos S. Tlemissov, Galina Antonova, Andrej M. Grjibovski

**Affiliations:** 1 Department of Public Health, Semey State Medical University, Semey 071400, Kazakhstan; marzick85@mail.ru (M.A.D.); yumsem@mail.ru (Y.M.S.); kail_galiya@mail.ru (G.K.); gulmanabaeva@mail.ru (G.K.M.); bakyt-64t@mail.ru (B.T.); 20indira04@mail.ru (M.S.Z.); oksanayrk@mail.ru (O.A.Y.); aidos_8668@mail.ru (A.S.T.); ssmu.univ@gmail.com (G.A.); 2 Central Scientific Research Laboratory, Northern State Medical University, 163000 Arkhangelsk, Russia; 3 Department of Public Health, Health Care, Hygiene and Bioethics, North-Eastern Federal University, 677000 Yakutsk, Russia

**Keywords:** antenatal care, satisfaction, clients, determinants, Kazakhstan, Central Asia

## Abstract

Women’s satisfaction is a part of the quality assurance process with potential to improve antenatal health services. The objective of this study was to assess the prevalence of women’s satisfaction with antenatal care in an urban Kazakhstani setting and investigate associated factors. A total of 1496 women who delivered in all maternity clinics from 6 February through 11 July 2013 in Semey, East Kazakhstan, filled out a standardized pretested questionnaire on satisfaction with antenatal care. Independent associations between dissatisfaction and its correlates were studied by logistic regression. Ninety percent of the women were satisfied with the antenatal care. Women who were dissatisfied had lower education. These women would have preferred more checkups, shorter intervals between checkups, more time with care providers, and shorter waiting times. The overall dissatisfaction was associated with long waiting times and insufficient information on general health in pregnancy, results of laboratory tests, treatment during pregnancy, and breastfeeding. Although most of the women in the study setting were satisfied with the new antenatal care model, we identified the main sources of dissatisfaction that should be addressed. Given that Semey is a typical Kazakhstani city, the results can be generalized to other Kazakhstani urban settings.

## 1. Introduction

Most cases of maternal and perinatal mortality occur in women who receive no antenatal care with more than 99% of these women living in developing countries [1]. Those countries which were part of the former Soviet Union inherited the old Soviet system of antenatal care, which was accessible and affordable for nearly all pregnant women. At the same time, most pregnancies were classified as “high risk” with between 15 and 30 antenatal visits during pregnancy and frequent hospitalizations. The treatment was not considered to be evidence-based and women’s satisfaction was not prioritized [2,3,4,5].

Shortly after breakup of the Soviet Union, the use of antenatal care services in the former Soviet Republics in Central Asia declined during the 1990s with a shift towards giving birth at home [6,7]. Considerable social inequalities in the use of antenatal services, limited knowledge about possible complications during pregnancy and childbirth among women, and limited capacity and knowledge of service providers have been consistently reported by international teams that promoted safe motherhood in the area [8,9].

Contrary to the other Central Asian states, 99.2% of Kazakhstani women attend a skilled antenatal care provider at least once during pregnancy. Antenatal care is provided primarily by doctors (82.6%), but also by midwives (15.3%), and in rare cases by other health professionals [10]. A pilot project on the introduction of international approaches to safe motherhood was conducted in Zhezkazgan in 2002–2003. It included a shift towards less-medicalized, more women- or family-centered care and more evidence-based approaches to antenatal care [11]. The main results of the project included a reduction in the number of prenatal visits per woman from 12 to 6, decline in the number of hospitalizations, reduction in the average length of stay in the maternity units, and a reduction in the use of non-evidence-based interventions, all leading to the fact that 98% of the women in Zheskazgan were satisfied by the care they received [11].

A nationwide reform of antenatal care had been gradually introduced between 2007 and 2010. A new Edict from the Ministry of Health regulating a reduction of the number of antenatal visits, use of evidence-based practice, and more personalized attitudes towards antenatal care was issued 7 April 2010. Maternal mortality in Kazakhstan decreased from 37.2 per 100,000 live births in 2009 to 13.6 per 100,000 live births in 2013 [12]. However, the quality of care provided by maternal institutions assessed in 2013 was considered substandard [13]. At the same time, no information on whether Kazakhstani women are satisfied and accept the new antenatal care is available in peer-reviewed literature. 

Client satisfaction is an integral part of the quality assurance process and client feedback has been shown to be useful to improving health service delivery [5]. International studies on the determinants of satisfaction by antenatal care yield controversial results warranting studies in other settings [14,15,16,17,18,19,20]. Evaluations of women’s overall satisfaction by the simplified evidence-based model of care with reduced number of antenatal visits has concluded that this model was well accepted by the women and had no long-term consequences for mothers and their children [21,22,23,24,25,26]. However, it has been emphasized that any changes in the delivery of antenatal care services should take into account women’s opinions [22]. A recent qualitative study from Kazakhstan has suggested that cultural and historical aspects should be considered when adopting international models of care [27], but quantitative estimates of women’s satisfaction and its determinants in Kazakhstan remain unknown.

The aim of this study was to assess women’s satisfaction with antenatal care and associated factors in an urban Kazakhstani setting.

## 2. Materials and Methods

The study was conducted in the town of Semey (former Semipalatinsk), East Kazakhstan, which is one of the main industrial towns in the country and is known for being the Soviet nuclear weapon testing site in 1949–1991. The population of Semey was 335,400 in 2013. Obstetric services in the town are performed in two municipal maternity hospitals and a perinatal center covering the total population of Semey and adjacent rural areas. Antenatal care is provided at policlinics. Outpatient antenatal services as well as the obstetric care are free of charge. 

A total of 1506 women who delivered in all municipal maternity clinics from February 6 to July 11, 2013 comprised the initial sample. Five (0.33%) of them moved to the town prior to delivery and were excluded from the study because they did not receive antenatal care in Semey. Moreover, five (0.33%) women with serious complications during delivery refused to participate in the study. Thus, the final sample consisted of 1496 women or 99.3% of the women who gave birth in Semey during the study period. Data about maternal age, parity, date of delivery, and date of the last menstrual period as well as the number of antenatal visits were obtained from the medical files.

Maternal satisfaction with the antenatal care was assessed using a 24-item questionnaire administered within three days after birth while the woman was in the maternity facility. Maternity clinics were selected as data collection sites because they do not provide antenatal care, minimizing the probability of social desirability bias. The questionnaire was developed by the WHO and used for research purposes in other countries [20,22,23]. The questionnaire was translated from English into Kazakh and Russian languages and back to ensure correctness of the translation. The Kazakh and Russian versions were then pretested on the personnel of the Department of Obstetrics and Gynecology, Semey State Medical University, and on a sample of pregnant women in one of the policlinics. Only minor changes were introduced into the formulation of the questions. Moreover, questions on education, income, ethnic background, and place of residence were added. For the purpose of this study, we used 15 questions on women’s preferences on the number of antenatal visits, waiting time, and time spent with the caregiver, as well as amount and appropriateness of the information received during the visits. Given previously reported questionable validity of questions related to satisfaction with antenatal services, we used only one question (“In general, how satisfied are you with the antenatal care you have received?”) to synthesize women’s overall perceptions of the quality of antenatal care [28]. 

Women were divided into three age groups: <20 years, 20–29 years, and 30 years or older. Maternal education was classified as secondary, vocational, and higher. Income per family member was coded as below 20,000 tenge (KZT, 1 USD~330 KZT), 20,000–29,999 KZT, 30,000–49,999 KZT, and 50,000 KZT or more. The level of 20,000 KZT was considered as the poverty line in Kazakhstan in 2013. The other cut-off points correspond to the second and the third quartiles of the income distribution. Three groups were used for women’s ethnic background: Kazakhs, Russians, and others. By parity, women were classified as primipara, women with 1 previous delivery, and 2 or more previous deliveries. Number of antenatal visits was dichotomized into 7 or less and 8 or more visits. Gestational age was calculated in weeks starting from the first day of the last menstruation period. Preterm birth was birth before 37 completed gestational weeks.

The overall level of satisfaction by antenatal care in the original questionnaire was coded as “very satisfied“, “satisfied”, and “not satisfied”.

Continuous data were presented as means (M) and standard deviations (SD). Proportions of women who were satisfied with antenatal care were presented with 95% confidence intervals (CI) calculated using Wilson’s method [29], which is considered to be superior to the most commonly used Wald. Bivariate relationships were assessed by Pearson’s chi-squared test. Associations between independent variables and dissatisfaction with antenatal care were studied using multivariable logistic regression. Crude and adjusted odds ratios (OR) with 95% CI were calculated. The reference group for each of the variables was chosen to be what is believed to be the most favorable category. A backward elimination procedure was applied to obtain the final regression model. This method is preferable to the forward method which runs a greater risk of Type II error and has a greater statistical power than the forced entry method for the given sample size [30]. Statistical Package for the Social Sciences (SPSS, version 17.0, SPSS Inc., Chicago, IL, USA) was used for all analyses.

The study was approved by the ethical committee of the Semey State Medical University (Protocol 2 from 20 November 2012, Project Identification Code). All women were informed about the aims of the study and signed informed consent forms.

## 3. Results

Altogether, 30.1% of the respondents were very satisfied while 59.9% were satisfied with antenatal care. As in most other studies, we dichotomized the outcome as satisfied (“satisfied” and “very satisfied” combined) and not satisfied for further analyses to ensure international comparisons.

In our sample, most of the women were 20–29 years old, had higher education, and were ethnic Kazakhs. Primiparous women comprised nearly half of the sample. More than a quarter of the women reported their income to be below the poverty line. Altogether, 5.6% of infants in the sample were born preterm. The number of antenatal visits varied from 1 to 18 (M = 7.5, SD = 2.6). Nearly half of the women had 7 or less checkups during the index pregnancy. Socio-demographic characteristics of the sample are presented in Table 1.

Altogether, 90.0% (95% CI: 88.3–91.4) of the women were satisfied with antenatal services. No associations between any of the socio-demographic characteristics and dissatisfaction with antenatal care were observed in bivariate analyses (Table 1).

The reported waiting time at the clinic before being seen by the antenatal care provider varied between 0 and 300 min with an average of 35.7 min. For further analyses the waiting time was categorized into three groups: 0–59 min, 60–119 min, and 120 min or more. Altogether, 22.6% of the women had to wait for more than 2 h before being seen by a medical professional. The reported time usually spent with the antenatal care provider ranged from 3 to 60 min with a mean of 19.8 min (SD = 9.3). For further analyses the time spent with the care provider was classified into three categories: <15 min, 15–44 min, and 45 min or more. Both waiting time and time with the providers of antenatal care were significantly associated with dissatisfaction in crude analyses (Table 2). 

Women who were dissatisfied with antenatal care were more likely to have preferred more checkups, and reported that the number of checkups was less than expected and that the time between checkups was too long (*p* < 0.001 for all questions). Moreover, they were more likely to be unhappy with the waiting time and would have preferred more time with the care provider (*p* < 0.001 for both questions). No difference between the satisfaction with the antenatal care and either the gender of the provider or his/her professional background within the given categories was observed (Table 2). 

Altogether, 35.9% of the women have reported that they received too little or no information on family planning. The corresponding proportions for the information about breastfeeding, labor, treatment during the index pregnancy, looking after own health, and various tests (blood, urine, etc.) were 30.5%, 25.8%, 13.7%, 11.7%, and 10.0%, respectively. All questions related to the information given to the women were significantly associated with the satisfaction with the antenatal care in crude analysis (Table 3).

In adjusted analysis, the only socio-demographic variable associated with satisfaction with the antenatal care was maternal education—women with secondary or vocational education were more likely to be dissatisfied than women with higher education. Women who were dissatisfied with the care would have preferred more checkups, more time with the antenatal care provider, and shorter intervals between checkups. Moreover, the overall dissatisfaction was associated with the time they had to wait and with either insufficient or no information on looking after own health, tests in the index pregnancy, treatment during this pregnancy, and breastfeeding. Women who had to wait for 60–119 min or did not remember whether they received information on family planning were more likely to be satisfied with the antenatal care than the reference categories (Table 4).

## 4. Discussion

This is, to the best of our knowledge, the first study on the satisfaction of Kazakhstani women with antenatal services after the new women-oriented antenatal care was introduced in Kazakhstan. 

Our findings are generally in line with the results of most of the studies conducted in developing countries which have shown that most of the women are satisfied with antenatal care [25]. However, this proportion is lower than what was reported from the pilot site in Zheskazgan in 2005 [11]. This may be explained by the fact that the pilot study was conducted in close cooperation with international agencies and the local health providers were more enthusiastic about new routines compared with the situation in Semey. 

Social variations have been shown to influence the level of satisfaction in some settings [17] but not all [14,26]. Positive associations between satisfaction and maternal age, parity, and education have been observed in several developing settings and were explained by more experience and better utilization of services by older, multiparous, and better educated women [25]. This may also be true for the association between education and satisfaction observed in this study, although no associations with other socio-demographic factors such as age, parity, ethnic background, or income were found.

Promptness of care and time spent with health care provider has been consistently shown to be among the most important factors for satisfaction with antenatal services [25]. Similar to in other studies, women who were dissatisfied with antenatal care reported that they were unhappy with the waiting time, and would have preferred to have more time with the provider, more checkups, and shorter intervals between checkups [14,19,28]. At the same time, women who had to wait between 1 and 2 h were more likely to be satisfied than women who waited for less than an hour in our study. This may be partly explained by the general perception among women that a thorough checkup should take time and long queues can reflect providers’ popularity, careful filling out of documentation, etc., thus warranting a qualitative study to explain this unexpected finding. It is interesting that dissatisfied women wished more often to be seen by a combination of different health professionals (30.7% vs. 23.7% for those satisfied). Although the difference did not reach the level of statistical significance, the reasons behind this are worth studying.

Another significant source of dissatisfaction in our study is insufficient information the women receive from their providers about their own health, laboratory tests, treatment, and breastfeeding. Provision of cognitive support has been considered a critical determinant of satisfaction in maternity care in several countries [13,24,26]. While insufficient information in countries with a considerable proportion of foreign providers and high prevalence of illiteracy among women can be attributed to language barriers [14], this is unlikely to be the case in Kazakhstan where virtually everyone speaks Russian and many speak both Russian and Kazakh. The observed high prevalence of either insufficient or even no information on several important aspects related to maternal health should be of concern for the health authorities in Semey. 

Our findings should be interpreted with caution taking into account strengths and potential weaknesses of the cross-sectional study design [31]. The relatively large sample size if compared to many similar surveys from developing settings is an advantage that allows detection of more factors associated with the outcome. Consecutive inclusion of all women who delivered in the municipal maternity facilities over the specified period is another strength reducing the probability of sampling bias. It is unlikely that the general level of satisfaction with antenatal services or associations between the satisfaction and selected predictors will vary across seasons in Kazakhstan. Even if so, our study included a part of the cold season and a part of the warm season, reducing the probability of seasonal bias. The questionnaire was previously validated and used in other countries allowing comparability of the findings. Moreover, its Russian and Kazakh versions used in this study were back-translated into English with nearly a perfect match. In addition, they were pretested both on health professionals and on pregnant women. Antenatal care is provided at policlinics while the data on satisfaction with antenatal care were intentionally collected at maternity clinics after delivery to avoid social desirability bias. Policlinics and maternity clinics are two different types of institutions in the Kazakhstani healthcare system, ensuring elimination of social desirability or fear of retaliation. Moreover, this survey was performed by trained interviewers unrelated to medical personnel at the clinics. The participants were assured that the questionnaires were anonymous and the data were treated confidentially by the research team. Thus, fear of retaliation or simple social desirability is very unlikely.

However, the study excluded women with severe complications during delivery, which could have influenced the overall level of satisfaction. Women with unfavorable pregnancy outcomes are less likely to report being satisfied with maternity care [32]. Thus, our estimates of the general satisfaction may be overestimated, although given the fact that we excluded only 5 women, the degree of overestimation is very small. When we repeated our analyses for term pregnancy only, the coefficients changed only marginally and did not influence any of the results. Another limitation is that we studied women’s satisfaction in only one town, reducing generalizability of the findings. However, Semey is similar to most middle-sized Kazakhstani towns in terms of socio-economic characteristics of the population and the quality of healthcare, allowing extrapolation of our findings to comparable settings. At the same time, we do not recommend generalization of our results to rural areas where socio-demographic characteristics of the population as well as availability of antenatal care are different from in the urban areas.

## 5. Conclusions

Most women are satisfied with antenatal care in the study setting. Main sources of dissatisfaction have been identified. While dissatisfaction with the number of visits and longer spacing between them can be solved by better information about safety of these new routines for women without complications, a considerable proportion of women who do not receive sufficient information about various aspects of maternal health should be addressed by training of the health providers in health communication. 

## Figures and Tables

**Table 1:** Bivariate associations between satisfaction by antenatal care and maternal socio-demographic and obstetric characteristics.

Variables	*N* (%)	Satisfied, *N* (%)	Not Satisfied, *N* (%)	*p*
Age, years				0.310
<20	96 (6.4)	85 (6.3)	11 (7.3)	
20–29	1025 (68.5)	916 (68.1)	109 (72.7)	
30+	375 (25.1)	345 (25.6)	30 (20.0)	
Education				0.231
Secondary	277 (18.5)	247 (18.4)	30 (20.0)	
Vocational	543 (36.3)	481 (35.7)	62 (41.3)	
Higher	676 (45.2)	618 (45.9)	58 (38.7)	
Income, KZT				0.209
<20,000	440 (29.4)	388 (28.8)	52 (34.6)	
20,000–29,999	359 (24.0)	332 (24.7)	27 (18.0)	
30,000–49,999	436 (29.1)	389 (28.9)	47 (31.3)	
50,000+	261 (17.4)	237 (17.6)	24 (16.0)	
Ethnic background				0.625
Kazakh	1124 (75.1)	1014 (75.3)	110 (73.3)	
Russian	305 (20.4)	274 (20.4)	31 (20.7)	
Other	67 (4.5)	58 (4.3)	9 (6.0)	
Parity				0.084
0	681 (45.5)	606 (45.0)	75 (50.0)	
1	478 (32.0)	426 (31.6)	52 (34.7)	
2+	337 (22.5)	314 (23.3)	23 (15.3)	
Gestational age, weeks				0.087
<37	84 (5.6)	71 (5.3)	13 (8.7)	
37 or more	1412 (94.4)	1275 (94.7)	137 (91.3)	
Number of visits				0.655
1–7	734 (49.1)	663 (49.3)	71 (47.3)	
8 or more	762 (50.9)	683 (50.7)	79 (52.7)	
Total	1496 (100.0)	1346 (100.0)	150 (100.0)	

**Table 2:** Bivariate associations between satisfaction by antenatal care and answers to the questions on the antenatal visits.

Questions	*N* (%)	Satisfied, *N* (%)	Not Satisfied, *N* (%)	*p*
Are you happy about the number of antenatal checkups you have had, or would you have preferred:				
more checkups	23 (15.8)	190 (14.1)	47 (31.3)	<0.001
fewer checkups	60 (4.0)	52 (3.9)	8 (5.3)	
number of checkups was right	1199 (90.1)	1104 (82.0)	95 (63.3)	
Have the number of antenatal checkups been:				
More than you expected	186 (12.4)	172 (12.8)	14 (9.3)	<0.001
Less than you expected	205 (13.7)	154 (11.4)	51 (34.0)	
About the same as you expected	1105 (73.9)	1020 (75.8)	85 (56.7)	
Has the time between checkups been:				<0.001
Too short	107 (7.2)	97 (7.2)	10 (6.7)	
Too long	153 (10.2)	110 (8.2)	43 (28.7)	
About right	1236 (82.6)	1139 (84.6)	97 (64.7)	
How long do you usually have to wait at the clinic before being seen by a doctor/nurse/midwife who provides you antenatal care?				<0.001
0–59 min	1158 (77.4)	1058 (78.6)	100 (66.7)	
60–119 min	248 (16.6)	219 (16.3)	29 (19.3)	
120 or more min	90 (6.0)	69 (5.1)	21 (14.0)	
Are you happy with the time you normally have to wait?				<0.001
No	485 (32.4)	397 (29.5)	88 (58.7)	
Yes	1011 (67.6)	949 (70.5)	62 (41.3)	
How much time do you usually spend with the doctor/nurse/midwife who provides you antenatal care?				0.005
<15 min	334 (22.3)	285 (21.2)	49 (32.7)	
15–44 min	1137 (76.0)	1039 (77.2)	98 (65.3)	
45 min or more	25 (1.7)	22 (1.6)	3(2.0)	
Do you have enough time with the doctor/nurse/midwife during your checkups, or would you prefer:				<0.001
A lot more time	175 (11.7)	146 (10.8)	29 (19.3)	
A little more time	198 (13.2)	158 (11.7)	40 (26.7)	
Time is about right	1123 (75.1)	1042 (77.4)	81 (54.0)	
If you had a choice, would you prefer to be seen by:				0.090
A male provider	78 (5.2)	66 (4.9)	12(8.0)	
A female provider	825 (55.1)	753 (55.9)	72(48.0)	
No preference	593 (39.6)	527 (39.2)	66(44.0)	
If you had a choice, would you prefer to be attended by:				0.189
A doctor	832 (55.6)	754 (56.0)	78 (52.0)	
A nurse	10 (0.7)	10 (0.7)	0 (0.0)	
A midwife	136 (9.1)	127(9.4)	9 (6.0)	
A combination	365 (24.4)	319(23.7)	46 (30.7)	
No preference	153 (10.2)	136(10.1)	17 (11.3)	

**Table 3:** Bivariate associations between satisfaction with antenatal care and answers to the questions on the information received in antenatal care.

Questions	*N* (%)	Satisfied, *N* (%)	Not Satisfied, *N* (%)	*p*
Was the information you received about looking after your own health:				<0.001
Not enough	144 (9.6)	95 (7.1)	49 (32.7)	
As much as you wanted	1135 (75.9)	1050 (78.0)	85 (56.7)	
Too much	87 (5.8)	84 (6.2)	3 (2.0)	
No information received	32 (2.1)	24 (1.8)	8 (5.3)	
Don’t remember	98 (6.6)	93 (6.9)	5 (3.3)	
Was the information you received about tests (e.g., blood, urine) during this pregnancy:				<0.001
Not enough	92 (6.1)	53 (3.9)	39 (26.0)	
As much as you wanted	1190 (79.5)	1100 (81.7)	90 (60.0)	
Too much	99 (6.6)	96 (7.1)	3 (2.0)	
No information received	58 (3.9)	43 (3.2)	15 (10.0)	
Don’t remember	57 (3.8)	54 (4.0)	3 (2.0)	
Was the information you received about any treatment you might need during this pregnancy:				<0.001
Not enough	92 (6.1)	60 (4.5)	32 (21.3)	
As much as you wanted	1098 (73.4)	1016 (75.5)	82 (54.7)	
Too much	73 (4.9)	71 (5.3)	2 (1.3)	
No information received	114 (7.6)	90 (6.7)	24 (16.0)	
Don’t remember	119 (8.0)	109 (8.1)	10 (6.7)	
Was the information you received about labor:				<0.001
Not enough	149 (10.0)	112 (8.3)	37 (24.7)	
As much as you wanted	938 (62.7)	877 (65.2)	61 (40.7)	
Too much	101 (6.8)	99 (7.4)	2 (1.3)	
No information received	237 (15.8)	189 (14.0)	48 (32.0)	
Don’t remember	71 (4.7)	69 (5.1)	2 (1.3)	
Was the information you received about breastfeeding:				<0.001
Not enough	121 (8.1)	92 (6.8)	29 (19.3)	
As much as you wanted	829 (55.4)	793 (58.9)	36 (24.0)	
Too much	148 (9.9)	141 (10.5)	7 (4.7)	
No information received	334 (22.4)	261 (19.4)	73 (48.7)	
Don’t remember	64 (4.3)	59 (4.4)	5 (3.3)	
Was the information you received about family planning:				<0.001
Not enough	84 (5.6)	71 (5.3)	13 (8.7)	
As much as you wanted	728 (48.6)	683 (50.7)	45 (30.0)	
Too much	105 (7.0)	97 (7.2)	8 (5.3)	
No information received	454 (30.3)	374 (27.8)	80 (53.3)	
Don’t remember	125 (8.4)	121 (9.0)	4 (2.7)	

**Table 4:** Results of multivariable logistic regression analysis.

Questions	aOR	95% CI
How long do you usually have wait at the clinic before being seen by a doctor/nurse/midwife who provides you antenatal care?		
0–59 min	1.00	Reference
60–119 min	0.52	0.28–0.96
120 or more min	1.57	0.77–3.21
Education		
Secondary	1.87	1.07–3.27
Vocational	1.66	1.04–2.63
Higher	1.00	Reference
Was the information you received about looking after your own health		
Not enough	1.82	1.04–3.20
As much as you wanted	1.00	Reference
Too much	0.58	0.15–2.25
No information received	1.13	0.37–3.42
Don’t remember	0.69	0.24–1.94
Was the information you received about tests (e.g., blood, urine) during this pregnancy		
Not enough	4.77	2.63–8.68
As much as you wanted	1.00	Reference
Too much	0.55	0.16–1.95
No information received	3.64	1.72–7.73
Don’t remember	0.68	0.18–2.59
Was the information you received about any treatment you might need during this pregnancy		
Not enough	2.19	1.17–4.09
As much as you wanted	1.00	Reference
Too much	0.26	0.05–1.36
No information received	1.48	0.78–2.81
Don’t remember	0.88	0.38–2.05
Was the information you received about breastfeeding		
Not enough	4.82	2.52–9.25
As much as you wanted	1.00	Reference
Too much	0.97	0.36–2.63
No information received	3.22	1.93–5.38
Don’t remember	1.67	0.53–5.38
Was the information you received about family planning		
Not enough	0.78	0.32–1.85
As much as you wanted	1.00	Reference
Too much	1.47	0.54–3.99
No information received	1.42	0.88–2.29
Don’t remember	0.22	0.06–0.79
Are you happy about the number of antenatal checkups you have had, or would you have preferred		
More checkups	1.96	1.17–3.28
Fewer checkups	0.97	0.38–2.48
Number of checkups was right	1.00	Reference
Has the time between checkups been		
Too short	0.50	0.21–1.16
Too long	2.28	1.32–3.94
About right	1.00	Reference
Are you happy with the time you normally have to wait?		
No	2.44	1.51–3.96
Yes	1.00	Reference
Do you have enough time with the doctor/nurse/midwife during your checkups, or would you prefer		
A lot more time	1.48	0.84–2.61
A little more time	1.84	1.10–3.07
Time is about right	1.00	Reference

## References

[1] World Health Organization (WHO) (2003). Antenatal Care in Developing Countries. Promises, Achievements and Missed Opportunities. An Analysis of Trends, Levels and Differentials.

[2] Chalmers B. (2005). Maternity care in the former Soviet Union. Br. J. Obstet. Gynaecol..

[3] Chalmers B., Quliyeva D. (2004). A report of women’s birth experiences in Baku, Azerbaijan. J. Psychosom. Obstet. Gynaecol..

[4] Dennis L.I., Flynn B.C., Martin J.B. (1995). Characteristics of pregnant women, utilization, and satisfaction with prenatal services in St. Petersburg, Russia. Public Health Nurs..

[5] Ivanov L.L., Flynn B.C. (1999). Utilization and satisfaction with prenatal care services. West. J. Nurs. Res..

[6] Kamiya Y. (2011). Women’s autonomy and reproductive health care utilisation: Empirical evidence from Tajikistan. Health Policy.

[7] Fan L., Habibov N.N. (2009). Determinants of accessibility and affordability of health care in post-socialist Tajikistan: Evidence and policy options. Glob. Public Health.

[8] Wiegers T.A., Boerma W.G., de Haan O. (2010). Maternity care and birth preparedness in rural Kyrgyzstan and Tajikistan. Sex. Reprod. Healthc..

[9] Falkingham J. (2003). Inequality and changes in women’s use of maternal health-care services in Tajikistan. Stud. Fam. Plan..

[10] Smailov A.A. (2012). Multiple Indicator Cluster Survey (MICS) in the Republic of Kazakhstan 2010–2011. Monitoring the Situation of Children and Women.

[11] (2005). Introducing International Approaches to Safe Motherhood in Zheskazgan: Results of a Pilot Project in Kazakhstan.

[12] World Health Organization Health for All Database. http://data.euro.who.int/hfadb.

[13] Dauletyarova M., Semenova Y., Kaylubaeva G., Manabaeva G., Khismetova Z., Akilzhanova Z., Tussupkaliev A., Orazgaliyeva Z. (2016). Are women of East Kazakhstan satisfied with the quality of maternity care? Implementing the WHO tool to assess the quality of hospital services. Ir. J. Public Health.

[14] Ghobashi M., Khandekar R. (2008). Satisfaction among Expectant Mothers with Antenatal Care Services in the Musandam Region of Oman. Sult. Qaboos Univ. Med. J..

[15] Richard L., Séguin L., Champagne F., Therrien R. (1992). Determinants of satisfaction with medical prenatal care in Quebec women. Can. J. Public Health.

[16] Fawole A.O., Okunlola M.A., Adekunle A.O. (2008). Clients’ perceptions of the quality of antenatal care. J. Natl. Med. Assoc..

[17] Esimai O.A., Omoniyi-Esan G.O. (2009). Wait time and service satisfaction at Antenatal Clinic, Obafemi Awolowo University Ile-Ife. East Afr. J. Public Health.

[18] Hundley V., Rennie A.M., Fitzmaurice A., Graham W., van Teijlingen E., Penney G. (2000). A national survey of women’s views of their maternity care in Scotland. Midwifery.

[19] Langer A., Villar J., Romero M., Nigenda G., Piaggio G., Kuchaisit C., Rojas G., Al-Osimi M., Belizán J., Farnot U. (2002). Are women and providers satisfied with antenatal care? Views on a standard and a simplified, evidence-based model of care in four developing countries. BMC Women’s Health.

[20] Sikorski J., Wilson J., Clement S., Das S., Smeeton N. (1996). A randomised controlled trial comparing two schedules of antenatal visits: The antenatal care project. Br. Med. J..

[21] Nigenda G., Langer A., Kuchaisit C., Romero M., Rojas G., Al-Osimy M., Villar J., Garcia J., Al-Mazrou Y., Ba’aqeel H. (2003). Womens’ opinions on antenatal care in developing countries: Results of a study in Cuba, Thailand, Saudi Arabia and Argentina. BMC Public Health.

[22] Langer A., Nigenda G., Romero M., Rojas G., Kuchaisit C., al-Osimi M., Orozco E. (1998). Conceptual bases and methodology for the evaluation of women’s and providers’ perception of the quality of antenatal care in the WHO Antenatal Care Randomised Controlled Trial. Paediatr. Perinat. Epidemiol..

[23] Clement S., Candy B., Sikorski J., Wilson J., Smeeton N. (1999). Does reducing the frequency of routine antenatal visits have long term effects? Follow up of participants in a randomised controlled trial. Br. J. Obstet. Gynaecol..

[24] Clement S., Sikorski J., Wilson J., Das S., Smeeton N. (1996). Women’s satisfaction with traditional and reduced antenatal visit schedules. Midwifery.

[25] Srivastava A., Avan B.I., Rajbangshi P., Bhattacharyya S. (2015). Determinants of women’s satisfaction with maternal health care: A review of literature from developing countries. BMC Pregnancy Childbirth.

[26] Craig B.J., Kabylbekova Z. (2015). Culture and Maternity Care in Kazakhstan: What New Mothers Expected. Health Care Women Int..

[27] Oladapo O.T., Osiberu M.O. (2009). Do sociodemographic characteristics of pregnant women determine their perception of antenatal care quality?. Matern. Child J..

[28] Das P., Basu M., Tikadar T., Biswas G., Mridha P., Pal R. (2010). Client satisfaction on maternal and child health services in rural Bengal. Indian J. Community Med..

[29] Grjibovski A.M. (2008). Confidence intervals for proportions. Ekol. Cheloveka.

[30] Field A. (2005). Discovering Statistics Using IBM SPSS Statistics.

[31] Kholmatova K.K., Grjibovski A.M. (2016). Cross-sectional studies: Planning, sample size and data analysis. Ekol. Cheloveka.

[32] Cham M., Sundby J., Vangen S. (2009). Availability and quality of emergency obstetric care in Gambia’s main referral hospital: Women-users’ testimonies. Reprod. Health.

